# Renal Denervation in Heart Failure Treatment: Data for a Self-Fulfilling Prophecy

**DOI:** 10.3390/jcm13226656

**Published:** 2024-11-06

**Authors:** Kyriakos Dimitriadis, Panagiotis Iliakis, Nikolaos Pyrpyris, Fotis Tatakis, Christos Fragkoulis, Vasileios Mantziaris, Aristides Plaitis, Eirini Beneki, Panagiotis Tsioufis, Dagmara Hering, Anastasios Kollias, Dimitrios Konstantinidis, Konstantinos Tsioufis

**Affiliations:** 1 First Department of Cardiology, School of Medicine, National and Kapodistrian University of Athens, Hippokration General Hospital, 115 27 Athens, Greece; panayiotisiliakis@gmail.com (P.I.); npyrpyris@gmail.com (N.P.); fotistatakis@yahoo.gr (F.T.); christosfragoulis@yahoo.com (C.F.); man.basilis@gmail.com (V.M.); aris.plaitis@gmail.com (A.P.); e.beneki@hotmail.com (E.B.); ptsioufis@gmail.com (P.T.); kon_dimitris@hotmail.com (D.K.); ktsioufis@gmail.com (K.T.); 2 Department of Hypertension and Diabetology, Medical University of Gdansk, 80-214 Gdansk, Poland; dagmara.hering@uwa.edu.au; 3 Hypertension Center STRIDE-7, School of Medicine, Third Department of Medicine, National and Kapodistrian University of Athens, Sotiria Hospital, 115 27 Athens, Greece; taskollias@gmail.com

**Keywords:** hypertension, heart failure, autonomous nervous system, renal denervation, neuromodulation

## Abstract

Renal denervation (RDN), a transcatheter renal sympathetic nerve ablation procedure, is a relatively novel established procedure for the treatment of hypertension, with it being recognized as a third option for hypertension management in the most recent European guidelines, together with pharmacotherapy, for achieving blood pressure targets. Given the relationship between both hypertension and sympathetic overdrive and the development of heart failure (HF), even studies at the dawn of research on RDN explored it as a treatment to overcome diuretic resistance in those patients. As it is now recognized that RDN does not only have organ-specific but also systemic effects, several investigators have aimed to delineate whether renal sympathetic denervation could alter the prognosis, symptoms, and adverse events of HF patients. Data are available in both HF patients with reduced and preserved ejection fraction. As the significance of neuromodulation is gaining grounds in the HF therapeutic arsenal, in this review, we aim to provide a rationale for using RDN in HF and an up-to-date overview of available data in both HF phenotypes, as well as discuss the future of neuromodulatory therapy in HF management.

## 1. Introduction

Heart failure (HF), according to the 2021 European Society of Cardiology (ESC) Guidelines for the diagnosis and treatment of acute and chronic heart failure, is defined as a clinical syndrome that includes both characteristic symptoms (dyspnea, fatigue, and ankle swelling) and signs (increased jugular venous pressure, peripheral oedema, and pulmonary crackles), and it is the pathophysiological result of often simultaneous alterations in both structural and functional characteristics of the heart, reflected as inadequacy to maintain cardiac output [1]. Traditionally, according to left ventricular ejection fraction (LVEF), HF can be divided into HF with reduced ejection fraction (HFrEF–LVEF ≤ 40%), HF with mildly reduced ejection fraction (HFmrEF–LVEF 41–49%), and HF with preserved ejection fraction (HFpEF–LVEF ≥ 50%). New York Heart Association (NYHA) functional classification is the easiest and most common clinical classification of the functional status of patients with HF. Regarding HF epidemiology, the overall prevalence of HF is rising to 1–3% of the total adult population globally, which is attributed to the better diagnosis of HFpEF patients more recently, as better diagnostic algorithms have been established [2]. Furthermore, mortality due to HF remains high, as it has been shown that the 30-day mortality rate is ~2–3%, while the 5-year mortality rate reaches ~50–70%, after hospitalization for HF [2]. Although there are established pharmaceutical treatment strategies proven to reduce cardiovascular mortality due to HF, further research, development, and evaluation of novel treatment modalities are needed [1,2,3].

Sympathetic overdrive is present in the entire spectrum of cardiovascular disease [4,5]. In hypertension, sympathetic nervous system (SNS) overactivation not only plays an important role in the pathophysiological cascade of disease development and progression but has also been the target of both pharmaceutical and interventional treatment [6,7,8]. Pharmaceutical treatment is mainly represented by β-blockers, while interventional treatment is represented by renal denervation (RDN). After almost 20 years of research, breakthroughs, and setbacks, large and long-term randomized controlled trials (RCT), and meta-analyses, RDN is recommended as a treatment option for patients with uncontrolled hypertension despite a triple blood pressure lowering combination or patients with increased cardiovascular risk and uncontrolled hypertension on fewer than three drugs. This has been clearly elucidated in both the 2023 European Society of Hypertension (ESH) and 2024 ESC guidelines of hypertension [7,8]. With hypertension being one of the most common risk factors for the development and progression of HF and the established common relationship, including the overactivation of the SNS in both entities, it is rational to hypothesize that RDN could be an efficient therapeutic modality in our arsenal toward battling HF [9]. Recently, there has been a lot of interest in research regarding the role of attenuating SNS overdrive in HF settings, as well as novel pharmaceutical and interventional treatment in HF [10,11]. There have been many animal studies on both hypertension and HF [12,13]. The feasibility, safety, and potential efficacy of RDN for HF and other cardiometabolic comorbidities have been suggested and studied many years ago [14]. Moreover, poor or low medication adherence—about 27–50% of patients with HF do not optimally adhere to their prescribed medications—plays a key role in HF disease progression and a potential target group of patients that are either non-tolerant or non-adherent to β-blockers could be candidates for RDN [15,16,17]. Given the pleiotropic effects of RDN, this review aims to delve into SNS involvement in HF pathophysiology, provide evidence regarding SNS as a potential therapeutic target in HF patients, and specifically examine the safety and efficacy of RDN in both preclinical and clinical trials in this population.

## 2. Sympathetic Overdrive in HF

### 2.1. Pathophysiology of Imbalance of the SNS

The involvement of the autonomic nervous system (ANS), especially the distortion of equilibrium between sympathetic and parasympathetic activity, plays a key role in the pathophysiology of HF [5]. It is established knowledge that in normal cardiac function, SNS is associated with a great number of cardiovascular actions, such as a rise in heart rate (HR), contractility increase, a reduction in venous vasculature capacitance, and an increase in peripheral vessel resistance; the parasympathetic nervous system (PNS), on the other hand, is responsible for the opposite actions [5]. Regarding nerves’ anatomy, sympathetic nerve fibers are in the subepicardium, while parasympathetic fibers, originating mainly from the vagus nerve, are located in the subendocardium [18]. The first are mainly presented in the ventricular tissue, while the latter are mainly in the atrial tissue. It is well known that noradrenaline (NA) is the main transmitter of the SNS, binding to specific receptors; human cardiac tissue mainly contains β1, β2, and β3 receptors [19]. The activation of these receptors results in the aforementioned actions of the SNS.

HF is the result of multiple structural, mechanical, and/or electrical abnormalities, with them most commonly being the result of myocardium injury, caused by coronary artery disease, hypertension, valvular dysfunction, and/or tachyarrhythmias [20]. It is of great importance to understand that several mechanisms are simultaneously involved in the pathophysiological cascade leading to the failing heart, including increased hemodynamic overload, neurohumoral overdrive, impaired cellular activity, inflammation, and apoptosis of cardiac myocytes and fibrosis [21]. Systolic and diastolic dysfunction often occur at the same time or overlap; in systolic dysfunction, the main substrate regards the loss of myocardial tissue, leading to contractility impairment, an increase in left ventricular end-diastolic (LVEDD) and end-systolic diameter (LVESD), and an increase in left ventricular end-diastolic pressure (LVEDP), eventually resulting in a decrease in stroke volume (SV) and LVEF [22]. Regarding diastolic dysfunction, myocardial contractility is usually preserved, with no imminent effect on LVEF, though there is impairment in early diastolic relaxation of LV and increased stiffness of both atrial and ventricular cavities, resulting in an increase in LVEDP, leading to increased venous congestion and eventual consequences for the atrial myocardium, which then leads to an increase in atrial dimension, usually associated with the increased presence of atrial fibrillation [23,24]. Finally, SNS overdrive is strongly associated with metabolic alterations in the entire spectrum of cardiovascular disease [25,26].

In all types and stages of HF, activation of the SNS seems to be an early response to all pathophysiological changes and triggers [21]. The aim of this activation in all settings is the preservation of SV and cardiac output to provide normal blood flow to all human tissue. Although at earlier stages, there is overactivation of b-receptors, leading to increased contractility, left ventricular hypertrophy, and increased peripheral resistance, at later stages, SNS overdrive is connected to a down-regulation of β1-receptors, with decreased responsiveness to adrenergic stimulation, evidence of NA-derived apoptosis and abnormal cardiac reflexes [27,28]. In the context of HF pathophysiology, it is of great importance to understand cardiovascular reflexes and observed abnormalities in their function. SNS activity is mediated by a series of reflex triggers and responses, which in HF seem to be dysfunction [5]: impaired baroreflex HR control in response to BP changes, impaired cardiopulmonary reflexes (inability of pulmonary and atrial receptors to inhibit SNS overdrive in response to volume overload), and dysfunction of peripheral chemoreceptors. Regarding cardiorenal reflexes, in HF, there is an inability to inhibit SNS efferent activation at the kidney level. In addition, part of the cardiorenal reflexes is the association between renal efferent sympathetic activity and atrial natriuretic peptide (ANP) levels in a functional antagonism model with ANP levels [29]. Moreover, at the renal level, it is evident that SNS overdrive leads to exaggerated activation of the renin–angiotensin–aldosterone axis, primarily via increasing renin release and decreasing renal perfusion, resulting in increased salt and water retention that leads to increased preload that further deteriorates diastolic dysfunction by increasing LVEDD and LVEDD [30]. The latter seems to be associated with a poorer prognostic impact in HF disease and progression [31].

### 2.2. Measurement of SNS Tone

It is of great importance not only to understand how autonomic imbalance plays a crucial role in HF progression but also to measure this sympathetic overdrive, which mainly aids in following up novel treatment modalities [32]. We will refer to the most common modalities for evaluating SNS activity, as well as the advantages and limitations of each marker (Table 1).

#### 2.2.1. Plasma and Urine Noradrenaline (NA) Levels

It is established knowledge that blood and urine NA levels are increased in HF and are associated with poorer prognosis [33,34,35]. This was also shown by Conn et al., who, almost 30 years ago, demonstrated a strong association in a multivariate analysis between increased NA levels and the subsequent risk of mortality [36]. This was also verified in the V-HEFT II study, which highlighted the association between NA levels over 900 pg/mL and greater mortality risk [37]. NA turnover can be assessed by imaging modalities, including organ-specific techniques, that use radio-labeled guanethidine analogs of NA [38]. Elevated NA levels reflect sympathetic overdrive in the failing heart [39]. Regarding organ-specific NA evaluation, it has been found that NA spillover measured from the heart is increased up to 50 times compared to other tissues in HF [40]. Moreover, it has been shown that increased NA levels not only play a crucial role in chronic HF but also mediate it via an endogenous catecholamine surge, the clinical manifestation of acute HF [41]. However, the fact that medical treatment shows heterogenous outcomes regarding reducing NA levels and the high-expertise laboratory equipment and experienced operators required limit its use in research settings, making it less available and feasible for daily clinical routine [42,43].

#### 2.2.2. Microneurography

Microneurography is an accurate method of quantifying sympathetic nerve activity in the muscles (MSNA) or the skin (SSNA) and is measured in either burst frequency/min or burst per 100 heartbeats [44,45]. In 1986, Leimbach et al. were the first to assess and evaluate muscle sympathetic activity and its relationship to circulating norepinephrine levels (NE) in patients with HF [46]. Indeed, they demonstrated in 16 patients with moderate–severe HF that MSNA levels were significantly higher compared to healthy control subjects (54 ± 5 vs. 25 ± 4 bursts/min, *p* < 0.01) and the presence of a strong correlation between MSNA and NE levels; more importantly, both MSNA and NE levels seemed to be positively correlated with LV filling pressure and mean right atrial pressure. Badrov et al. compared MSNA between patients with HF and healthy controls, demonstrating that mean MSNA, adjusted for age, sex, BMI, and HR, was greater in HF patients(+14.2 bursts/min; 95% confidence interval [CI] 12.1–16.3; *p* < 0.0001) [47]. Regarding prognosis, MSNA values higher than 49 burst/min seem to be independently associated with HF mortality [33]. Furthermore, pharmacological treatment of HF, and specifically after 6 months of carvedilol, is related to the reduction in MSNA, which shows the importance of this marker (not only does it increase in the presence of the disease, but it also decreases on grounds of successful treatment) [48]. It is of great importance to note that exercise training is associated with a reduction in MSNA in patients with HF, suggesting the importance of exercise in ameliorating sympathetic tone and improving cardiovascular health, as shown in a meta-analysis of 40 trials with 1253 patients [49]. In HFpEF, although dynamic exercise is associated with increased sympathetic tone, as evaluated based on MSNA, during static exercise, patients with HFpEF exhibited similar sympathetic activity to healthy controls, with the results showing firstly that dynamic exercise limits blood flow and increases peripheral resistance in this population and secondly that there are other factors that determine static exercise lower workload observed in HFpEF [50]. There are also few data from MSNA studies supporting that phenotype-guided treatment especially in sub-populations with HF and sleep apnea might benefit more from battling sympathetic overdrive [51]. However, we should appreciate that a major limitation of MSNA is that only efferent sympathetic signals can be recorded [44]; moreover, MSNA, being invasive, non-repeatable, and with a required learning curve, is currently restricted for research purposes and not utilized in the daily routine of the assessment of HF patients.

#### 2.2.3. Heart Rate and Heart Rate Variability (HRV)

Increased HR rate, as a result of an imbalance in ANS equilibrium, is shown to be an independent risk factor in the development and progression of HF. This was elucidated in the Rotterdam study that included 4768 subjects, without prevalent HF, who were followed up for almost 15 years; in total, 656 of them developed clinical HF. The investigators demonstrated that for every 10 beats/min increase in HR, the multivariate hazard ratio for the development of new-onset HF was significantly higher, at 1.16 (*p* = 0.005) [52]. In the emblematic SHIFT trial, patients with HF and mean HR > 75 beats/min were randomized to either ivabradine treatment (*n* = 3241) or matching placebo (*n* = 3264); it was shown that the ivabradine arm was associated with incremental reductions in both mortality and hospitalizations due to HF, highlighting the relationship between HR decrease and HF prognosis [53]. However, there are limitations in the utilization of HR as a marker of SNS activity, mainly its high variability that can be attributed to age, exercise, resting state, inflammation, temperature, etc.; furthermore, increased HR cannot distinguish the activation of SNS or the withdrawal of parasympathetic tone [54,55].

Heart rate variability refers to the fluctuation in the time intervals between adjacent heartbeats and quantifies the variations in RR (or NN) intervals in consecutive heartbeats [56]. HF is characterized by decreased HRV [55]. Moreover, HRV has been associated with the different HF subtypes, with low HRV being associated with an elevated risk of HFpEF, where little association was found with the risk of HFrEF in a longitudinal study of 28,603 individuals, with a mean follow-up of 17 years, that evaluated the connection between HRV and the prevalence of HF in post-menopausal women [57]. In the UK-Heart Study, Nolan et al. prospectively enrolled and followed up 433 HF patients, demonstrating that the standard deviation of NN intervals (SDNN), a marker of HRV, was significantly reduced and was proven to be the most powerful mortality predictor in a mean follow-up period of 14 months [58]. In CIBS (Cardiac Insufficiency Bisoprolol Study), a landmark trial of b-blockers in the field of HF, Pousset et al. demonstrated that bisoprolol 5 mg once daily was strongly associated with a significant improvement (increase) in HRV indices, compared to the matching-placebo arm [59]. Again, the inability to distinguish whether sympathetic overdrive or parasympathetic withdrawal contributes more to HRV changes in the HF population, as well as the variability in prognostic results, slightly restrict its use in daily clinical routine.

#### 2.2.4. Baroreceptor Sensitivity (BRS)

Although this review focuses on SNS overdrive in HF disease progression, we should appreciate baroreceptor sensitivity (BRS) as a marker of parasympathetic activity [60]. BRS refers to the ability of both carotid and aortic baroreceptors to recognize, adapt, and respond to acute or chronic BP changes. In HF, parasympathetic tone is slightly or significantly reduced, reflected in depressed BRS, and studies have shown that it is associated with poorer prognosis in this population, and regarding treatment, b-blockers seem to improve BRS activity [61]. There are numerous both invasive and non-invasive modalities to assess BRS activity, although there is no standardized method regarding its efficacy. Of them, HR turbulence seems to be the more accurate indirect measure and surrogate of BRS [60]. It relies on the counting and analysis of all ventricular ectopic beats captured on a 24 h Holter; its first limitation is that it cannot be applied in the presence of atrial fibrillation. In the MUSIC (Muerte Subita en Insuficiencia Cardiaca) trial, Iwona Cygankiewicz et al. enrolled 607 HF patients who were followed up for 44 months; the investigators demonstrated that abnormal HR turbulence was strongly associated with all-cause mortality in patients with QRS > 120 ms [62]. In a more recent study, Charytan et al. evaluated BRS activity in patients with HF and chronic kidney disease (CKD), demonstrating that BRS and CKD were independently associated, and moreover, depressed BRS impacted cardiovascular mortality, independently of CKD presence [63].

## 3. Preclinical Data on RDN in HF

In the last 40 years, there has been a great deal of interest not only in how sympathetic overdrive affects the genesis of HF but also in finding and evaluating novel techniques for ameliorating its impact on HF symptoms and prognosis. In this direction, several preclinical studies have been performed, as a precursor in establishing human trials [64].

### 3.1. Renal Physiology

One of the first animal studies to evaluate the effect of RDN on HF was conducted by Kon et al. in 1985 [65]. They studied the effect of renal nerves in renal vasomotor tone, before and after surgical RDN in three groups of anesthetized Munich–Wistar rats: rats with HF after surgically induced myocardial infarction (group 1, *n* = 10), rats with acute extracellular fluid volume depletion after deprivation of drinking water for 48 h (group 2, *n* = 8), and sham or non-treated control rats (group 3, *n* = 6). They demonstrated that RDN was associated with a 36% increase in glomerular plasma flow rate, an increase in single nephron glomerular filtration rate, and a decrease in efferent arteriolar resistance in infarcted HF rats compared to the controls, highlighting a direct positive effect of RDN in this setting [66].

Mizelle et al. studied the association between renal nerve activity and sodium retention in a canine model of congestive HF, induced by rapid ventricular pacing. The investigators enrolled 10 female dogs, undergoing unilateral surgical RDN and urinary bladder split, allowing simultaneous 24 h urinary collection from both the denervated and innervated kidney. Although sodium excretion was decreased, there were no differences in renal hemodynamics or electrolyte excretion between innervated and denervated kidneys [67]. However, the short period of HF (only during the pacing period) and the fact that structural and functional changes in the kidneys due to HF often need more time to be established may have influenced these outcomes. In contrast, other investigators demonstrated that renal sympathetic activity plays an important role in sodium retention in HF animal models [68].

Furthermore, considering the role of ANP levels and renal sympathetic nerves, Pettersson et al. evaluated the effect of RDN on this relationship in ischaemic HF rats and showed that RDN was associated not only with reduced renal NA spillover but also with complete restoration of renal adaptation to ANP increases [69]. Villareal et al., in a dog model with high-output HF, validated this hypothesis, as an increase in sodium retention was reported post-RDN, followed by regaining sodium equilibrium [70].

With respect to sodium excretion, Villareal et al. conducted another study with high-output HF dogs, undergoing RDN, showing that RDN was associated with beneficiary effects in both total postprandial urinary and fractional sodium excretion, compared to innervated controls (*p* < 0.05), highlighting the attenuation of the expression of postprandial natriuretic mechanisms [71]. The effect of RDN on autoregulation of renal blood flow was further studied by DiBona et al., demonstrating in a model of rats with normal sympathetic activity and increased sympathetic activity (HF and hypertensive rats) that RDN was associated with an increase in renal blood flow variability in the latter [72], while Clayton et al., in pacing-induced HF, demonstrated that despite HF being strongly associated with a decrease in mean renal blood flow and increase in renal vascular resistance, both did not change following RDN. However, at the cellular level, RDN was associated with near normalization in the expression of angiotensin AT1 receptors, suggesting a potential explanation of the beneficiary renal hydrodynamic effects of RDN on the kidneys [73].

### 3.2. Cardiovascular Physiology

Following the positive effects of RDN on renal physiology, there was even greater interest in the role of RDN in LV geometry and function in the failing heart. Nozawa et al. conducted a study with myocardial infarction-induced HF rats, with half of them undergoing bilateral surgical denervation 2 days before infarction induction. They evaluated left ventricular function 4 weeks later in the two groups. They demonstrated that rats that had undergone RDN had lower LV end-diastolic pressure, significantly decreased LV end-diastolic and end-systolic diameter, and decreased sodium excretion, compared to controls (HF rats that did not undergo RDN) [74].

Regarding the high burden of arrhythmia in HF, it has been shown in dogs with pacing-induced HF that RDN is associated with significant attenuation of the ventricular substrate and regression of ventricular remodeling in HF [75]. Hu et al. evaluated the effect of RDN in left ventricular mechanical desynchrony in HF dogs, demonstrating higher LVEF, higher left ventricular global longitudinal strain, and lower levels of dyssynchrony compared to untreated HF dogs [76]. Similarly, Luo et al. suggested that RDN may lead to significant attenuation of ventricular electrical remodeling, alongside having potential anti-fibrillatory effects on the ventricular substrate and reducing ventricular fibrillation burden in a canine model of pacing-induced HF [77]. The aforementioned effects on reducing arrythmiological burden and ameliorating the risk of ventricular fibrillation were even more prominent when RDN was performed in the combined presence of obesity and HF [78]. Furthermore, RDN was also associated with a reverse of atrial remodeling in the HF canine model, which is known to lead to atrial fibrillation [79], as Wang et al. demonstrated that when RDN was compared to a control in a dog model with HF, it was significantly associated with a reduction in atrial fibrillation inducibility [80]. To gain a better understanding of sympathetic involvement in arrhythmogenicity, Yamada et al. demonstrated that RDN significantly reduced the bi-atrial effective refractory period in a rabbit HF model [81].

Moving on from the positive intermediate outcomes of RDN in animals with HF, Hu et al. compared the efficacy of surgical RDN vs. pharmacological treatment in rats with post-MI-induced HF, showing that when compared to beta-blockers, ACEIs, and ARBs, RDN was associated with significantly better cardiac remodeling and function, improved sodium excretion, and significant benefit in markers of autonomic tone [82]. When comparing RDN with beta-blockers in HF rats, RDN was able to slow down the progression of both myocardial and renal injury, similar to b-blockers. The overall effects were similar to those of b-receptor blockade [83]. Similar results in both the heart and kidneys were shown by Liu et al. in isoproterenol-induced HF rats, randomized to RDN and sham operation, demonstrating that RDN not only led to a reduction in NE and aldosterone levels, with simultaneous RAAS receptor downregulation, but also seemed to be associated with significant inhibition of both cardiac and renal fibrosis [84].

The positive results of surgical RDN as well as the positive results efficacy of catheter-based RDN in both animal and human settings [85,86] led Chen et al. to design and conduct an animal trial, enrolling HF dogs in either catheter-based RDN or sham-operation, showing that it is a safe and feasible procedure, without any procedure-related serious adverse events, and also that it is associated with both a significant reduction in HF biomarkers and significant improvement in left ventricular size and function (lower LVESD and LVEDD and higher LVEF; *p* < 0.05) [87]. Pinkham et al. demonstrated similar results in an MI-induced HF rat model [88]. An emblematic study by Polhemus et al. enrolled spontaneously hypertensive rats and normotensive rats that were subjected to MI-induced HF and underwent catheter-based RDN or sham-RDN 4 weeks later, resulting in significant improvement in LV function, mainly attributed to RDN-derived inhibition of renal neprilysin activity. The investigators highlighted the crucial role of renal nerve activity and, therefore, its blockade led to the attenuation of the neprilysin effect on kidney function, the augmentation of circulating NE levels, and a reduction in fibrosis [89]. Another key study demonstrated that catheter-based RDN, in MI-HF rats, was associated with inhibition of the renin–angiotensin system, an increase in circulating B-type natriuretic peptide levels, the attenuation of fibrosis, and improvement of LV function. Interestingly, RDN seemed to significantly improve coronary artery responses to vasodilators compared to controls [90].

Finally, when considering the effect of RDN as add-on to GLP-1 agonists for the treatment of HF [91], Katsurada et al. demonstrated in an HF rat model that selective afferent RDN is associated with a significant increase in diuretic and natriuretic responses to GLP-1 (urine flow 96.0 ± 1.9 vs. 53.4 ± 4.3 µL/min/gkw; sodium excretion 13.6 ± 1.4 vs. 7.4 ± 0.8 µEq/min/gkw) [92]. At a molecular level, Wang et al. highlighted the benefits of RDN in LV function and fibrosis amelioration, which mainly contributed to the downregulation of TGF-β/CTGF and upregulation of microRNAs: miR-29b, miR-30c, and miR-133a [93]. Regarding cellular and molecular changes, Wang et showed that RDN was strongly associated with a reduction in cardiomyocyte apoptosis [94]. Zheng et al. conducted one of the first studies to show that in the setting of HF, RDN leads to a significant reduction in the expression of epithelial sodium channels and aquaporin 2, which plays a key role in epithelial sodium channel function and sodium retention [95].

Lately, with the aim of delineating the intricate molecular background of these effects, Shen et al. highlighted that the improvement of LV function, alongside the reduction in sympathetic activity, is driven by repressing BACH1 and PACS-2-mediated mitochondrial oxidative stress by inactivating the TGF-β1/SMADs/SP1 pathway in a rat model of HF, expanding our understanding of the cellular-level benefits of SNS blockade in HF models [96].

## 4. Clinical Evidence

Moving forward from the positive results of preclinical studies and there being a well-documented pathophysiological relationship between HF and sympathetic overdrive, several investigators have aimed to evaluate whether RDN, along with the observed benefits of long-term BP control [97] and SNS activation reduction [98], also exerts any beneficial effect in HF in clinical settings. Already, studies including hypertensive patients have shown that RDN is associated with improvements in LVMI, circumferential strain, and LVEF [99,100]. Therefore, a number of observational, as well as randomized studies, have been performed, in order to test this hypothesis “from bench to bedside”.

### 4.1. Observational Studies

Davies et al. [101] were the first group to study the effects of RDN in HF patients. They included in their study seven patients on optimal medical therapy (OMT), in whom RDN was performed. At 6 months follow-up, the investigators did not find any RDN-related complications, thus documenting the method’s safety. In particular, no patients were admitted after the RDN procedure for HF symptoms or hypotensive/syncope episodes, while renal function remained stable throughout the 6-month follow-up period. All patients described an improvement of symptoms, while the 6 min walk test (6MWT) distance at 6 months was significantly increased (Δ = 27.1 ± 9.7 m, *p* = 0.03). No significant changes in echocardiographic parameters were reported, while regarding biochemical analysis, only a reduction in sodium levels of 3 mmol/L achieved statistical significance (*p* = 0.03). This study documents for the first time the safety of RDN in HF patients, while the improvement of symptoms and the 6 min walk test after the procedure was suggestive of benefit.

Following this, Gao et al. [102] studied 14 patients who underwent RDN and were followed up for 6 months. After RDN, no hypotensive episodes or syncope were reported at 6-month follow-up. Renal function, as determined by creatinine levels, also remained stable and was unaffected by RDN. LVEF was significantly increased from a baseline of 36.0 to 43.8% at the time of the follow-up (*p* = 0.003); however, no other echocardiographic markers were significantly different. Furthermore, BNP levels were found to be significantly reduced post RDN, from a baseline of 661.2 to 300.0 pg/mL (*p* = 0.008), while the 6MWT distance increased significantly from a baseline of 152.9 m to 334.3 m (*p* < 0.001).

Hopper et al. [103] further investigated the potential benefit of RDN in HFrEF patients. The study included 39 patients, which were already receiving OMT and were followed up for 12 months after the procedure. Only one safety event potentially linked to RDN was noted (renal artery occlusion), while six patients had a documented rise in creatinine levels of 25–50%. The authors reported no statistical significance in LVEF or 6MWT distance. However, they demonstrated a significant reduction in NT-proBNP levels (*p* = 0.006). Despite the fact that the only parameter indicative of benefit in this study was the reduction in NT-proBNP, the authors highlighted the lack of deterioration in HF status at 12 months, with this being indicative of a positive effect of RDN.

Kresoja et al. [104] retrospectively analyzed 99 HFpEF patients; in comparison with 65 non-HF patients undergoing RDN. Baseline characteristics suggested a higher SV and pulse pressure index in HFpEF patients compared to non-HF individuals, while they also observed lower aortic distensibility in the HF group. At the time of the analysis and following the RDN procedure, HFpEF patients showed a significant decrease in SV index (pre-RDN: 40 vs. post-RDN 37, *p* = 0.011) and increased aortic distensibility (pre-RDN: 1.5 vs. post-RDN: 1.7, *p* = 0.007). Furthermore, echocardiography showed that LV diastolic stiffness and LV filling pressures were also significantly decreased compared to baseline (*p* = 0.032 and 0.043 respectively), while there was a 24% decrease in NT-proBNP between baseline and after the procedure (*p* < 0.001). A follow-up analysis by the same author group showed that arterial elastance was reduced in both healthy and HFpEF patients (*p* < 0.01), while both end-systolic elastance and diastolic capacitance were significantly changed in HF but not in control patients [105].

Moreover, Rommel et al. [106] tried to investigate the effect of RDN on aortic stiffness in HFpEF by enrolling 60 patients (30 controls and 30 HFpEF patients) undergoing RDN and found that patients with HFpEF, at baseline, had increased rates of parameters associated with arterial stiffness. However, RDN resulted in significant benefits in these parameters, with increased total arterial compliance (mean difference (MD): 0.42; 0.17 to 0.67 mL/mm Hg), increased backward transit time normalized to LVEF (MD:1.7; 0.4% to 3.0%) and decreased reflection coefficient (MD: −2.6; −5.0% to −0.3%), accompanied by improvement in HF symptoms.

Aiming to understand the effect of RDN in different stages of HF, Geng et al. [107] studied 17 patients with HF who underwent RDN and were followed up for one year. Two subgroups were created based on the duration of HF, i.e., group 1 had an HF diagnosis of less or equal to 3 years (*n* = 9) and group 2 had an HF diagnosis of more than 2 years (*n* = 8). No RDN-related safety concerns were reported by the investigators throughout the follow-up period, i.e., no events of renal function deterioration, renal artery stenosis/dissection, or orthostatic hypotension. Importantly, there was a significant increase in LVEF when considering the total patient cohort (*p* < 0.05) and group 1 (*p* < 0.05) but not when examining only group 2. Left atrial and ventricular and right ventricular dimensions were also significantly improved in group 1. Interestingly, no group showed a significant change in BNP levels, while only the total cohort and group 1 showed a significant reduction in inflammatory biomarkers (TNF-a and CRP). Thus, these investigators note a potential greater advantage of RDN in patients at early stages of HF.

Finally, considering long-term results in patients with HFpEF, Vogt et al. [108] recently reported the 9-year follow-up outcomes of RDN in LV structure and function in HFpEF patients undergoing RDN. Out of 70 eligible patients, 21 had HFpEF. The investigators reported a significant reduction in HFA-PEFF score, from a baseline of 5.48 ± 0.51 points to 4.33 ± 1.53 points at 9 years (*p* < 0.01). This reduction was mostly associated with improvement in the morphological and biomarker subcategories (from 1.95 ± 0.22 to 1.43 ± 0.51 points and from 1.52 ± 0.52 to 0.90 ± 0.63 points, respectively; *p* < 0.01 for both) than in the functional one. However, the authors noted that the number of patients in the NYHA class greater or equal to two increased over the follow-up time period from two to six patients; however, this increase was non-significant, which could be explained by functional capacity limitations that acted later in the life of these patients. All observational trials are summarized in Table 2.

### 4.2. Randomized Controlled Trials

In light of evidence from early observational data, several randomized controlled studies have been performed in order to assess the efficacy and safety of RDN in HF patients (Table 3). Dai et al. [109] randomized 20 NYHA III-IV patients into two equal groups, undergoing either RDN or standard treatment. This study showed that, at 6 months follow-up, patients who underwent RDN had a significant increase in LVEF (RDN:45%; standard therapy: 38%; *p* < 0.001) as well as significantly lower BNP levels (RDN:424; OMT:604; *p* < 0.001), LV diameter (RDN:6.0; OMT:6.7; *p* < 0.001), and MACE (RDN:20%; OMT:80%; *p* = 0.024). Furthermore, the researchers reported that 24 h after the procedure, the RDN group had a significantly increased urine output, while levels of biochemical markers such as plasma renin, angiotensin II, aldosterone, BNP (*p* = 0.001), dopamine, adrenaline, and NA levels, as well as symptoms of dyspnea and edema, were found to be significantly lower. Regarding safety events, there was no reported arrhythmia related to RDN, renal failure defined by oliguria, or renal artery dissection.

Chen et al. [110] further studied the feasibility of RDN in HF patients in a randomized setting, enrolling 60 patients, who were randomly assigned in a 1:1 ratio to either undergo RDN in addition to optimal medical therapy or medically treated controls. Patients were followed up for 6 months. The RDN group had significantly increased LVEF at 6 months (RDN: 41.9%; OMT:31.2%; *p* < 0.001). Importantly, there was a slight decrease in LVEF reported in the OMT group (baseline 31.9%), while the LVEF of RDN patients increased from a baseline of 31.1% to 39.3% at 3 months and 41.9% at 6 months. Furthermore, patients who underwent RDN had improved 6MWT, compared to the OMT group, where no change was evident (*p* = 0.043). NYHA class was also significantly decreased following RDN, from 3.2 ± 0.5 to 1.6 ± 0.6 (*p* < 0.001). Finally, N-terminal pro–B-type natriuretic peptide (NT-proBNP) was also significantly decreased post RDN, compared with controls (*p* < 0.001). No safety events regarding the procedure, i.e., no artery stenosis or deteriorating renal function at 6 months, were noted.

Moving to HFpEF, Patel et al. [111] randomized 25 patients in a 2:1 fashion to either undergo RDN or remain in OMT. The primary efficacy endpoint was improvement in three of the six following parameters at 12 months follow-up: the Minnesota Living with Heart Failure Questionnaire (MLWHFQ); peak treadmill exercise oxygen uptake (VO2 peak); B-type natriuretic peptide (BNP); E/e′ (ratio of early mitral outflow velocity to average of medial and lateral mitral annular tissue velocity); left atrial volume index (LAVI); and LV mass index (LVMI). With respect to safety, two patients in the RDN arm had more than a 30% reduction in eGFR at 12 months. However, there were no reported renal artery stenoses while the median change in eGFR values was found to be similar between the two arms. The primary endpoint was not met and there were no significant improvements with RDN at 12-month follow-up, when the study was stopped due to the slow recruitment rate. However, an improvement in the composite efficacy endpoint was reported at 3 months in the RDN group, compared to the control arm (*p* = 0.018), mostly driven by significant changes in VO2 peak (56% vs. 13%, *p* = 0.025) and E/e′ (31% vs. 13%, *p* = 0.04). It is important to mention that this study was underpowered; therefore, it is susceptible to type II errors, and its results may not indicate the actual effects of RDN in HFpEF patients.

Following this, Drozdz et al. [112] evaluated the effect of RDN in patients with chronic HF and no response to cardiac resynchronization therapy (CRT). They included 20 patients with a median LVEF of 32.5%. These patients were randomly assigned in a 1:1 ratio to either receive RDN and OMT or OMT only. Interestingly, the investigators reported that RDN resulted in non-significant differences in LVEF, BP, 6MWT, and N-terminal prohormone of brain natriuretic peptide (NT-proBNP) levels at both 6 and 12 months after RDN. Similarly to previous trials, the procedure was safe, with no events of renal artery stenosis, renal artery dissection, pseudoaneurysm at the femoral artery access site, or bleeding being shown at 6- and 12-month follow-up.

Using a similar design to previous studies, Gao et al. [113] evaluated 60 patients who were randomly assigned to either RDN and medical therapy or medical therapy alone. The patients were NYHA class II-III and followed up for 6 months. In regard to the safety of the procedure, there were no reported hypotensive or syncope episodes nor a significant change in renal function at 6-month follow-up. The results of this study showed a significant decrease in NT-proBNP levels in the RDN group (RDN: 440.1; control: 790.8; *p* < 0.001). Also, the LVEF of patients in the RDN group was significantly higher in comparison to the OMT arm (RDN: 39.1%; control: 35.6%; *p* = 0.017), while LVEDD in the RDN group was significantly reduced compared to the control *p* (RDN: 46.4 mm; control: 50.2 mm; *p* < 0.001). Furthermore, the 6MWT distance was significantly increased in RDN patients (RDN: 301.2 m; control: 227.2 m; *p* = 0.01), while seven patients in the RDN group also showed an improvement in NYHA class.

More recently, Feyz et al. [114] performed the randomized IMPROVE-HF-I study, which randomly assigned 50 HFrEF patients in a 1:1 ratio to either RDN or OMT. At 6 months follow-up, the primary safety endpoint, which was defined as the combination of CV death, HF rehospitalization, and acute kidney injury, did not differ between groups (8.3 vs. 8.0%; *p* = 0.97). Moreover, eGFR remained unchanged in both the RDN and control groups. Regarding efficacy, the mean change in late iodine-123 meta-iodobenzylguanidine heart-to-mediastinum ratio (HMR) and washout rate was not significantly different between the groups (*p* = 0.95 and 0.09; respectively). Therefore, the study concluded that RDN does not result in changes in cardiac sympathetic nerve activity 6 months after the procedure in HF patients.

In patients with secondary HF due to Chagas disease, Spadaro et al. [115] performed a randomized study in order to assess the effects of RDN, including 17 patients, allocated to either RDN (11 patients) or OMT (6 patients). All patients had severely depressed left ventricular function, with a mean LVEF of 26.7 ± 4.9%. At 9-month follow-up, the composite of all-cause mortality, stroke, MI, need for renal artery intervention, or worsening renal function occurred in 36.4% of the RDN patients and 50% of the control arm, without a statistically significant difference (*p* = 0.60). No difference was also noted in laboratory, functional, or echocardiographic parameters after the intervention, therefore indicating a potential absence of RDN benefit in this patient subgroup. However, the low number of patients included should be taken into consideration and the results should be cautiously interpreted, while further studies are necessary in order to delineate the full effect of RDN in this patient subgroup. Finally, no in-hospital complications related to the procedure were noted in the study by the investigators, whilst the available laboratory studies show similar renal function between baseline and follow-up between the evaluated cohorts.

Finally, aiming to combine the results of RDN with respect to safety and efficacy in patients with HF, Su et al. [116] performed a meta-analysis in patients with HFrEF, including eight studies and 314 patients. The study showed that RDN was associated with a significant increase in LVEF (+9.59%; 95% CI: 7.92–11.27; *p* < 0.01); a decrease in BNP and NT-proBNP levels (*p* < 0.01 in both); and functional status improvement with significant benefits observed in NYHA classification and 6MWT distance. Significant improvement was also found in cardiac dimensions, with a mean reduction of 4 mm in LVEDD (*p* < 0.01) and 4.7 mm in LA diameter (*p* < 0.01). Corroborating these results, the meta-analysis of Li et al. [117] also found significant improvement in LVEF and 6MWT distance following RDN in HFrEF patients.

## 5. Clinical Perspectives

RDN is, from 2023, a guideline-endorsed treatment for hypertension. The initial recommendation was made in 2023 European Society of Hypertension (ESH) guidelines on the management of hypertension, where RDN was suggested as a treatment option for patients with true resistant or uncontrolled hypertension with a class II, level of evidence B recommendation [7]. Recently, the 2024 European Society of Cardiology (ESC) guidelines on the management of hypertension also recommend the use of RDN in a similar ESH cohort of patients, with a class IIb, level of evidence B recommendation [8]. Thus, the use of RDN in hypertension is now endorsed by societies, given not only its safety and efficacy but also the already mentioned consistent, long-term reductions in BP. Despite sympathetic overdrive being a common denominator in several cardiovascular pathologies, the use of RDN in them remains investigational. In this context, available preclinical and clinical data support the hypothesis that RDN exerts benefits in patients with HF, leading to augmented LVEF, decreased cardiac dimensions, and symptomatic improvement. Although not all studies found a significant correlation between RDN and HF improvement, the results of the aforementioned meta-analyses support, to date, the safety and efficacy of RDN in HF patients (Graphical Abstract). However, there is still a need for larger, randomized trials that will employ RDN in addition to the current optimal pharmacotherapy treatment, including ARNI and SGLT2 inhibitors, as several older trials did not include such regimens in their study protocol due to them not being recommended at the time of their respective study period. Moreover, as in hypertension, investigators should focus on identifying a distinct HF patient phenotype, which benefits the most from renal sympathetic nerve ablation, which could result in larger employment of this modality in clinical practice.

Currently, a number of trials evaluating the effect of RDN in HF are ongoing. The UNLOAD-HFpEF (NCT05030987) and RDN-HFpEF (NCT05715697) trials are two randomized trials that will further test the safety and efficacy of RDN in this patient phenotype. UNLOAD-HFpEF is anticipated to enroll 68 patients undergoing either RDN or remaining in OMT, with the primary outcome being exercise pulmonary capillary wedge pressure at 6-month follow-up. Other endpoints will include, among others, mortality, NYHA class, HF hospitalizations, and differences in biomarker levels at 6-, 12-, and 24-month follow-up. Similarly, RDN-HFpEF will enroll an estimated number of 200 patients with hypertension and HFpEF, who will be randomized to RDN or pharmacotherapy, with the primary outcome being the change from baseline of E/E’ at 12 months follow-up. Another study, RE-ADAPT-HF (NCT04947670), will enroll 144 patients with chronic HF, LVEF < 45%, and NYHA class II-III, who will also be randomized to either an RDN group or a control group. The primary outcome will be the change in 6MWT distance, while secondary outcomes will include functional and biochemical variables. The RESURRECT study (NCT05703620) is a non-randomized study that will examine the effect of RDN in several high-cardiovascular-risk patients and will also include patients with HFrEF (along with CKD and ESRD subgroups) and will compare the reduction in renal sympathetic nerve activity with the spillover and MSNA methods from baseline at 3 and 12 months, respectively, as well as changes to BP levels until 36 months of follow-up. Finally, another study (NCT04719637), enrolling 15 participants, will assess the safety and efficacy of ultrasound RDN in patients with HF, reporting MIBG cardiac washout rate as a primary and several functional and exercise parameters as secondary outcomes. Results from some of these studies are expected as early as 2025.

Besides RDN, several other neuromodulation systems have been studied in HF patients [11]. However, none is clearly suggested in HF guidelines. Notably, in 2019, a baroreceptor activation treatment (BAT) device gained FDA approval in patients with advanced HF, based on the BeaT-HF trial [118], which showed significant improvements in quality of life, exercise capacity, and levels of NT-proBNP, while a recent, independent patient data meta-analysis also supports the safety and efficacy of this device in patients with HFrEF [119]. Other interventions include vagus nerve stimulation, which aims to upregulate vagal nervous activity and thus restore ANS balance, which has reported conflicting results in clinical trials, however [120,121,122], which necessitate further investigations (NCT03425422). Another intervention, endovascular baroreflex activation, uses a stent implanted in the internal carotid artery, which alters carotid sinus shape and increases wall strain, thus increasing baroreflex activation. This intervention has also been used in hypertension treatment [123], with some limitations regarding changes in HR and pharmacotherapy adherence [124]. In HFrEF, this device showed that it was safe while demonstrating improvements in quality of life, 6MWT distance, and LVEF, as well as a 28% reduction in NT-proBNP levels [125]. Finally, several other neuromodulation devices such as splanchnic nerve stimulation and aortic thoracic neuromodulation have been tested in HF, with early positive results [126], but more research is needed in order to showcase clinical benefit.

As mentioned above, it should be noted that in the case of RDN evidently proving its benefit in HF patients in future randomized studies, the intervention will be accompanied by long-term medical treatment with the current pillars of pharmacotherapy for HF, which include already known neuromodulatory agents (beta-blockers), as well as the more recently established SGLT2 inhibitors, which show significant clinical benefit in both HFrEF and HFpEF, mostly due to their pleiotropic effects in cardiovascular homeostasis. The clinical, echocardiographic, and functional improvement of such patients following SGLT2 inhibitor initiation is well documented [127,128], while besides classic mechanisms considered to be cardioprotective [129], it has been recently reported that SGLT2 inhibitors, among other pathways, benefit the coronary microvasculature, which is evidently impaired in patients with HF, therefore contributing to enhanced myocardial perfusion [130]. This could translate not only into enhancement of cardiac mechanics and energetics but also to a substantial improvement in clinical symptoms, which has to be more extensively validated in future studies, Moreover, as with RDN, SGLT2 inhibitors show hints of neuromodulatory effects of the SNS. More specifically, recent studies, both preclinical and clinical, report conflicting results, with either a benefit or neutral effect of these agents in markers of sympathetic activation [131]. However, as there are several pathophysiological links that could explain SNS drive reduction with the use of SGLT2 inhibitors, future, more well-designed studies, are necessary in order to fully comprehend whether these agents could ultimately exhibit a benefit in HF-related autonomic dysfunction [131]. Finally, SGLT2 inhibitors are of benefit in arrhythmias and particularly AF, with a meta-analysis by Mariani et al. [132] indicating that dapagliflozin reduced the risk for AF in both the overall general population and patients with diabetes. Therefore, it is essential to determine whether a potential combination of pharmacotherapy and catheter-based treatment for HF could result in a greater benefit via interruption of multiple pathogenetic pathways, which could ultimately benefit the patient more. In that notion, more recent additions in the arsenal of HF treatment, such as GLP-1 agonists, are also being investigated in order to establish a link with the reduction in sympathetic overactivation, with early results showing that it could be a promising target in future investigations [133].

Finally, the benefit of RDN in HF is possibly not related only to improvements in cardiac mechanics and functional status *per se* but also to the pleiotropic effects of this intervention in cardiovascular physiology. To date, RDN has been reported to be of benefit in atrial arrhythmias burden, with reductions in atrial fibrillation (AF) burden and antiarrhythmic drug usage, when combined with pulmonary vein isolation (PVI) [134]. Similar results have been shown in a recent meta-analysis evaluating combined RDN and PVI, including seven trials and 711 patients, with sustained BP reductions and AF recurrence risk [135]. Furthermore, RDN has demonstrated significant improvements in the clinical severity of obstructive sleep apnea (OSA), along with BP reductions [136]. Finally, metabolic and glucose homeostasis, as also regulated by the SNS, has also been found to be improved by RDN, with mostly beneficial, but also inconsistent, results throughout the available literature [137]. All of these effects of RDN show promise in improving the overall cardiovascular phenotype of HF patients, thus reducing mortality and cardiovascular events in these high-risk cohorts.

## 6. Conclusions

In patients with HF, RDN has the potential to improve myocardial function, as shown by echocardiography indices, as well as symptom control. The pleiotropic effects of this intervention, along with the increasing evidence of benefit in HF phenotypes, set the ground for further, larger studies and randomized studies documenting novel indications for this procedure. Identifying the phenotype that would benefit more from neuromodulation in both clinical and symptomatic factors, together with current pharmacotherapy options, is essential for providing RDN as an option in patients with HF and beyond.

## Figures and Tables

**Table 1:** Strengths and limitations of SNS measurement modalities.

Measurement Modality	HF Prognosis	Advantages	Limitations
Noradrenaline (NA) spillover	NA levels over 900 pg/mL are associated with increased mortality	“Gold-standard” method Specific tissue examination Comparative evaluation of regional spillover	Heterogenous response to treatment High-expertise laboratory equipment Experienced operators
Microneurography (MSNA)	MSNA values over 49 burst/min are associated with increased mortality Pharmacological treatment of HF associated with MSNA reduction	Ability to evaluate central sympathetic outflow to the periphery both at rest and during exercise Utilized in a clinical trials’ setting for comparative treatment evaluation	Only efferent sympathetic signals can be recorded Invasive, non-repeatable, and required learning curve Limitations in the arrhythmogenic substrate
Heart rate Variability (HRV)	Decrease in SDNN (a marker of HRV) is significantly associated with increased mortality Pharmacological treatment with β-blockers is associated with HRV improvement	Non-invasive, easy-to-operate, and time-efficient method Provides both office and out-of-office real-life data	Inability to distinguish the impact of parasympathetic withdrawal vs. sympathetic overdrive High variability in prognosis prediction
Baroreceptor Sensitivity (BRS)	Depressed BRS lower than 3.0 ms/mmHg is associated with ↑ mortality Abnormal HR turbulence strongly is associated with all-cause mortality when QRS > 120 ms	Non-invasive and easy to operate	Marker of parasympathetic activity Variability of the method Non-applicable in the presence of atrial fibrillation

**Table 2:** Observational clinical studies of RDN in HF.

Author	Year	Study Type	N	Patient Characteristics	Follow-Up	Changes in Imaging Outcomes	Change in Laboratory Values	Changes in Functional Outcomes	Safety
Davies et al. [101]	2013	Observational	7	Chronic systolic HF	6 months	NR	NR	All patients experienced symptom improvement. 6MWT distance was significantly increased (Δ = 27.1 ± 9.7 m, *p* = 0.03)	No patient was admitted for HF symptoms or procedural complications. No hypotensive episodes or syncope events. Renal function remained stable throughout the follow-up
Gao et al. [102]	2017	Observational	14	Chronic HF, LVEF < 45%	6 months	LVEF increased from 36.0 ± 4.1% to 43.8 ± 7.9% (*p* = 0.003)	NR	The 6MWT distance increased from 152.9 ± 38.0 m to 334.3 ± 94.4 m (*p* < 0.001)	No hypotensive episodes or syncope was reported at 6 months follow-up. Creatinine levels remained stable
Hopper et al. [103]	2017	Observational	39	HFrEF	12 months	Non-significant change in LVEF (28 ± 9% vs. 29 ± 11%; *p* = 0.536)	Significant reduction in NT-proBNP (1530 ± 1228 vs. 1428 ± 1844 ng/mL; *p* = 0.006)	Non-significant change in 6MWT distance (384 ± 96 vs. 391 ± 97 m; *p* = 0.584)	One patents experienced a renal artery occlusion event that did not meet the protocol definition of stenosis. Six patients had a rise in creatinine from 25% to 50%
Kresoja et al. [104]	2021	Observational	164	HFpEF (*n* = 99) and healthy controls (*n* = 65)		Stroke volume index, LV diastolic stiffness, and LV filling pressures significantly decreased in HFpEF (*p* = 0.011, 0.032, and 0.043, respectively) Aortic distensibility (*p* = 0.007) and systolic stiffness (*p* < 0.001) increased	NT-proBNP significantly decreased in HFpEF patients (*p* < 0.001)	NR	NR
Fengler et al. [105]	2022	Observational	99	HFpEF, high LVEF (*n* = 63), and normal LVEF (*n* = 36)	NR	Ea was significantly reduced in both groups (*p* < 0.001) Ees/Ea was significantly increased in normal LVEF but not in high LVEF (*p* < 0.05)	NR	NR	NR
Geng et al. [107]	2018	Observational	17	HF, HF duration < 3 years (*n* = 9), and HF duration > 3 years (*n* = 8)	12 months	Significant increase in LVEF in the total cohort and <3 years duration (*p* < 0.05 for both) but not in >3 years	No significant change in BNP. Significant reduction in inflammatory markers in the total cohort and HF < 3 years	No significant change in 6MWT distance	No reported events of worsening renal function, renal artery stenosis/dissection, or orthostatic hypotension
Vogt et al. [108]	2024	Observational	21	HFpEF	9 years	Significant reduction in HFA-PEFF score (5.48 ± 0.51 to 4.33 ± 1.53 points; *p* < 0.01) Greater decrease in morphological and biomarker categories rather than functional. Concomitant reduction in LV hypertrophy			NR

HF: Heart Failure; 6MWT: 6 Minutes Walking Test; LVEF: Left Ventricular Ejection Fraction; HFrEF: Heart Failure with reduced Ejection Fraction; HFpEF: Heart Failure with Preserved Ejection Fraction; NT-proBNP: N Terminal—pro B-Type Natriuretic Peptide; BNP: Brain Natriuretic Peptide; LV: Left Ventricle; HFA-PEFF: Heart Failure Association Pre-test assessment, Echocardiography and Natriuretic Peptide Score, Functional testing, and Final etiology; NR: Not Reported.

**Table 3:** Randomized clinical studies of RDN in HF.

Author	Year	Study Type	N	Patient Characteristics	Follow-Up	Changes in Imaging Outcomes	Changes in Laboratory Outcomes	Changes in Clinical Outcomes	Safety
Dai et al. [109]	2015	RCT	20	HF and NYHA III-IV	6 months	Significantly increased LVEF in the RDN group (45 vs. 38%; *p* = 0.001)	Neurohormonal levels were decreased compared with pre-operation and controls	Symptomatic improvement in patients post RDN	No events of procedure-related arrhythmia, oliguria, or renal artery dissection were reported
Chen et al. [110]	2016	RCT	60 (1:1)	HF	6 months	Significant improvement in LVEF in the RDN group (*p* < 0.001)	Significant improvement in NT-proBNP (*p* < 0.001) in the RDN arm	Significant improvement in NYHA class (*p* < 0.001) and all domains of SF-36, except body pain (*p* = 0.74).	No artery stenosis at 6 months follow-up. Renal function remained stable
Patel et al. [111]	2016	RCT	25 (2:1)	HFpEF	3–12 months	Improvements in E/e’ (31% vs. 13%, *p* = 0.04) in those undergoing RDN	More patients improved at 3 months in the RDN arm regarding VO2 peak (56% vs. 13%, *p* = 0.025)	No significant change in the Minnesota Living with Heart Failure Questionnaire score	Two patients undergoing RDN had more than a 30% reduction in eGFR at 12 months. No reported new renal artery stenosis. Median change in eGFR was similar between RDN and controls
Drożdż et al. [112]	2019	RCT	20 (1:1)	HFrEF not responding to CRT	6 and 12 months	No significant differences in LVEF	No significant differences in BNP	No significant differences in 6MWT distance	No events of renal artery stenosis, renal artery dissection, pseudoaneurysm at the femoral access site or bleeding occurred at 6- and 12-month follow-up
Gao et al. [113]	2019	RCT	60 (1:1)	Chronic systolic HF	6 months	Significant increase in LVEF post-RDN (39.1 ± 7.3% vs. 35.6 ± 3.3%, *p* = 0.017)	Significant decrease in NT-proBNP (440.1 ± 226.5 pg/mL vs. 790.8 ± 287.0 pg/mL, *p* < 0.001) in the RDN arm	Significant improvement in NYHA class in those undergoing RDN (*p* = 0.01)	None reported hypotensive or syncope episode. No significant change of eGFR between groups at 6-month follow-up
Feyz et al. [114]	2022	RCT	50 (1:1)	HFrEF	6 months	No significant change in the composite of cardiovascular death, rehospitalization for HF, and acute kidney injury or change in iodine-123 meta-iodobenzylguanidine heart-to-mediastinum ratio at 6 months between groups			The composite safety endpoint of cardiovascular death, rehospitalization for heart failure, and acute kidney injury at 6 months was found similar between groups (8.3 vs. 8.0%). eGFR remained unchanged in both arms
Spadaro et al. [115]	2019	RCT	17 (2:1)	Chagas disease and HF	9 months	Similar echocardiographic parameters between groups at 9 months	NR	Similar functional/quality-of-life parameters between groups at 9 months	No in-hospital complications. No worsening renal function evident in follow-up laboratory values

HF: Heart Failure; 6MWT: 6 Minutes Walking Test; LVEF: Left Ventricular Ejection Fraction; HFrEF: Heart Failure with reduced Ejection Fraction; HFpEF: Heart Failure with Preserved Ejection Fraction; NT-proBNP: N Terminal—pro B-Type Natriuretic Peptide; LV: Left Ventricle; NR: Not Reported; NYHA: New York Heart Association; RDN: Renal Denervation; MACE: Major Adverse Cardiovascular Events.
